# Teaching Shared Decision Making to Undergraduate Medical Students

**DOI:** 10.5041/RMMJ.10453

**Published:** 2021-10-25

**Authors:** Meira Tidhar, Jochanan Benbassat

**Affiliations:** 1 Kibbutz Gvulot, Israel; 2 Department of Medicine (Retired), Hadassah University Medical Center, Jerusalem, Israel

**Keywords:** Communication skills, doctor, patient relations, medical education, patient counseling, patient preferences, shared decision making, undergraduate medical students

## Abstract

Despite the wide endorsement of shared decision making (SDM), its integration into clinical practice has been slow. In this paper, we suggest that this integration may be promoted by teaching SDM not only to residents and practicing physicians, but also to undergraduate medical students. The proposed teaching approach assumes that SDM requires effective doctor–patient communication; that such communication requires empathy; and that the doctor’s empathy requires an ability to identify the patient’s concerns. Therefore, we suggest shifting the focus of teaching SDM from how to convey health-related information to patients, to how to gain an insight into their concerns. In addition, we suggest subdividing SDM training into smaller, sequentially taught units, in order to help learners to elucidate the patient’s preferred role in decisions about her/his care, match the patient’s preferred involvement in these decisions, present choices, discuss uncertainty, and encourage patients to obtain a second opinion.

## INTRODUCTION

Since the 1970s, there has been a growing recognition of patients’ right to participate in decisions about their care, and many countries have legislated for patients’ informed consent to medical interventions. These developments promoted a shared decision making (SDM) consultation style, whereby patients convey their knowledge, concerns, and wishes about their problem, while doctors provide explanations about available courses of action.^1^ In theory, SDM should lead to better decision making, since decision aids have been claimed to increase patients’ risk perceptions and the number of decisions consistent with their values, and to reduce decisional conflict and the number of passive patients.^2^ Even though there is only weak evidence regarding health-related outcomes^3^ and quality of life,^4^ it is widely agreed today that the doctor should be able to apply SDM and reach an agreed course of action.^5^

Still, the adoption of SDM in clinical practice has been slower than that of patient-centered care. Since the 1970s, patient-centered care has evolved into a substantial component of undergraduate medical programs, and, already in the 2000s, various dimensions of patient-centeredness have been observed in about half of doctor–patient encounters.^6^,^7^ On the other hand, an analysis of doctor–patient interactions revealed that about 80% of SDM behaviors were patient-rather than doctor-initiated.^8^ A 2019 survey indicated that as many as 31% of doctors reported implementing a paternalistic approach in daily practice,^9^ and, as late as 2019, many doctors were unsure of the exact meaning of the related terms SDM, informed consent, risk assessment, and decision aids.^10^ Obviously, doctors need guidance in implementing SDM.

In this paper, we propose an SDM training program based on the view that, similar to patient-centered care, learning SDM may be improved if (a) it is included in undergraduate medical education, rather than offered to residents and practicing doctors only;^11^ and (b) its teaching emphasizes the ability of learners to understand patient concerns.

## APPROACHES TO TEACHING SHARED DECISION MAKING

Shared decision making first requires that doctors understand a patient’s need for information and are able to present that information in a way that empowers the patient to choose a course of action.^1^ Secondly, patients must want to participate in the decisions regarding their management and have an awareness of the uncertainty in medicine.^12^ Conventional wisdom defines the objectives of teaching SDM as (a) recognizing situations in which SDM is critical for a decision; (b) communicating to the patient the need for a decision; (c) describing the risks, benefits, and uncertainty associated with available options, often by using patient decision support; (d) understanding the patient’s preferences; and (e) reaching agreement regarding the patient’s management.^5^,^13^–^17^

To date, SDM teaching interventions aimed at achieving these objectives have emphasized how to communicate medical information and uncertainty by distributing printed materials, having educational meetings, and explaining the use of decision making aids.^18^,^19^ The effect of these interventions on learners’ knowledge, attitudes, and skills has been subject to several recent reviews of the literature. It has been found that all SDM training programs were implemented for residents and practicing doctors; that the reported trials were of poor quality; and that teaching SDM had little effect on the learners’ knowledge and comfort with SDM.^11^,^18^–^20^

Communicating management options and uncertainty, using patient decision support, eliciting patients’ preferences, and agreeing on management are certainly worthwhile objectives. However, we disagree with the recommended first two steps in SDM training, namely, recognizing when a decision is required and communicating the need for a decision to the patient.^5^,^13^–^17^ We believe that identifying the patient’s concerns is more important than acknowledging the need for a decision.

The teaching program that we propose is based on the following assumptions. Firstly, SDM training of undergraduate students should have more of an impact than teaching programs for residents and practicing doctors, just as the inclusion of training for patient-centered care into the undergraduate medical curriculum has led to patient-centeredness in about half of doctor–patient encounters.^6^,^7^ Secondly, we agree that SDM requires a patient-centered approach,^21^ which, in turn, requires empathy. Thirdly, we concur with the view that empathy is a multistep sequence that begins with understanding the patient’s concerns, and proceeds to emotional engagement, compassion, and the desire to help the patient.^22^ Finally, we believe that understanding the patient’s concerns is a teachable skill.^23^

## LEARNING OBJECTIVES OF SHARED DECISION MAKING

We propose defining the SDM learning objectives according to the following five statements.

During an encounter with a new patient or a patient with a new complaint, the student will:

1. understand the patient’s concerns, feelings, and sources of distress;
2. elucidate the patient’s preferred role in decision making;
3. match the patient’s preferred involvement in decision making;
4. present options and discuss pros, cons, and uncertainty;
5. offer the patient options for obtaining a second opinion and accessing reliable websites.

Each of these learning objectives is discussed below.

### Understanding Patient Concerns

In-depth interviews have indicated that patients expect their doctor to consider the specifics of their individual circumstances, and are disappointed when doctors present prognostic statistics only.^24^ To meet this expectation, doctors must not only fit the patient into a diagnostic category, but also find out what makes each patient unique by gaining insight into their specific concerns. Identifying patient concerns is the point of departure for both patient-centered care and SDM. It requires recognition that concerns may not be directly expressed.^25^ Patients seldom verbalize feelings spontaneously; rather, they offer clues and express their concerns only if encouraged to do so. Ignoring these clues may be the result of low tolerance for and little experience with negative emotional expression. For example, medical students are often mortified by a weeping patient and need reassurance that this is normal; they need to understand that the patient’s concerns are a legitimate subject of inquiry. Such an inquiry may include questions such as, *Of all that you told me, what makes you worry most? What do you want most to avoid? What are your plans for the future?* or *What do you expect from the treatment?*

### Elucidating the Patient’s Preferred Role

Doctor–patient relationships range from “paternalistic” (doctor decides, patient complies without any explanations) to “informative” (doctor provides information, patient decides), with varying degrees of patient involvement between these two extremes. Patients differ in their preferred role in their care. A 2012 systematic review revealed that even though most patients preferred to participate in decisions, there were those who wanted to delegate decisions. In studies conducted after 2000, most patients preferred SDM (71%) as compared to only 50% before 2000.^26^ Our 1998 literature review indicated that more than 92% of patients wanted to be informed about their illness. About half of them wanted information with a view to delegate decisions to their doctor, while the other half wanted to participate in their management. The remaining 3%–8% preferred a passive role without receiving any information.^27^

The variability of patient preferences for participation in decisions about their care is further complicated by their different attitudes to “information seeking,” “self-treatment,” and “involvement in clinical decisions.”^28^ The preferred involvement of patients in decisions about their own care differed from their involvement in the care of their children (Tidhar, 1998, unpublished MSc thesis) and from their preferred involvement in diagnostic problem solving.^29^ Female gender, white race, younger age, education, and income were all predictors of a patient’s preference to be involved in care.^30^ However, demographic characteristics explain only 25% of the variability in patient preferences for involvement in their care.^31^ This variability is not between patients only, but also within patients over time.^32^ Therefore, the only way a doctor can understand the preferences of individual patients for information and SDM is by direct enquiry.^30^,^32^

Poor and less educated patients commonly participate in fewer decisions about their care because doctors often underestimate their desire for information and ability to participate in SDM.^33^ Therefore, it is important to distinguish between patients who desire a passive relationship with their doctor, and those who are hesitant to ask questions but want to be involved in their treatment. To achieve this distinction, a doctor may ask: *Before I answer your questions, it would help if you told me what you already know about your disease*. The purpose of this question is to not only elucidate the patient’s understanding of his/her disease, but to also communicate an intent and willingness to respond to questions. Patients may answer by expressing their concerns (*I hope that it is a transient headache; however, I dread the possibility of cancer*), while other patients may not (*I don’t have the slightest idea*). In such cases, the doctor may invite again the patient to state his/her preferences by saying: *I am very interested to have your opinion about how we should proceed*, or *Do you want me to tell you my thoughts about your disease and the various options of its further investigation/treatment?* The patient’s answer (*Please just tell me what to do*, or *Yes, tell me what these options are*, or *Yes, tell me what do you think my disease is*) will probably clarify their preferences about involvement in the SDM process.

### Matching the Patient’s Preferred SDM Involvement

A 2006 literature review confirmed that most patients desire information about their illness, but that the desired information varies among patients. Its main conclusion was that doctors may optimize their encounters with patients by matching their needs for information and their preferences for involvement in treatment, rather than by strictly adhering to SDM.^34^ Patients may need information regarding their diagnosis (*What is the name of the disease? Is the diagnosis certain?*), need for treatment (*Why do I need an operation? Are there alternatives? What if I don’t have the operation?*), the effect of the disease (*Will I be a burden to the family? Will I suffer from pain?*), treatment (*How long will it take?*), or help in decision making (*Could you explain to me again …? What would you do if you were in my place?*). It is advisable to answer these questions honestly; to ascertain the patient’s preferences about sharing decisions with others (*Do you want to talk to me alone or in the presence of another person? Would you rather talk to someone in the family first?*); to avoid providing prognosis in terms of time; and, if necessary, to provide a broad realistic timeframe that would allow settling of personal affairs.

The patient’s needs are not limited to information. We mentioned earlier that the main predictors of patient satisfaction were his/her perception of the doctor as caring and sensitive. The way to convey the “I care” message is attentive listening, acceptance of expressions of emotions such as crying, and body language that transmits understanding and encouragement. In most cultures, this is done by eye contact and facing the patient without a table separating them.

### Presenting and Discussing Medical Options

Patient education is a cornerstone of SDM, and its main sources are doctors and the Internet. About half of chronically ill patients search the Web before consulting doctors in order to gather information about diagnosis, treatments, and specialists,^35^ and to make best use of the time available with their doctor.^36^ Nevertheless, most patients appear to trust their physician’s clinical expertise more than the Web.^35^

Evidence suggests that doctors find it difficult to share information with patients for two reasons. First, they may feel that acknowledging uncertainty would reduce patient trust and increase apprehension.^37^ However, this feeling is inconsistent with the view of bioethicists, that patients can manage information about uncertainty, that disclosure protects patient trust, and that SDM is particularly important in situations with substantial uncertainty.^38^ Second, communicating risks is difficult. It requires an understanding of the harms and benefits of an intervention, and skill to convey these risks to the patient. In response to evidence that many doctors are not up to this challenge, Koch et al.^39^ reported the successful implementation of a 15-hour teaching course that integrated basic statistics, bias detection, and communication skills. All of these topics had been taught separately during the undergraduate program; the objective of the teaching course proposed by Koch et al. was to reorganize previously acquired knowledge and apply it to SDM.

Finally, since patients need time to absorb the information received, it is important to schedule a follow-up meeting to provide additional clarification. The key question throughout the doctor–patient encounters would be: *Is there anything else you would like to know?* The role of the doctor is to be available; to be a continuous source of information and support while transmitting to the patient that expressions of grief, loss of hope, or anger are normal; and to be prepared to answer *I do not know* and absorb the patient’s anger.

### Offering a Second Opinion

The discrepancies between expert interpretations of imaging, histopathological, and clinical findings led to the agreement that all non-emergency patients are entitled to a second opinion.^40^ A 2016 literature review indicated that while the second opinion confirmed the previous one in 43%–82% of cases, in 12%–69% of cases it did not.^41^ The main barriers to seeking a second opinion were the patients’ sense of shock, time constraints, information overload, and concerns about the relationship with their doctor.^42^ Therefore, doctors’ encounters, particularly with patients who appear to be making decisions based on informal and idiosyncratic reasoning,^43^ should include encouragement to seek a second opinion (*Would you like to consult another doctor? Would you like to consult a doctor of your own choice?* or *Would you like me to recommend to you an expert?*) and reassurance that this will not affect future doctor–patient encounters (*I shall not be offended if you wish to have the opinion of another doctor; to the contrary—I shall be only too glad to have his/ her opinion about how to proceed*).

Patients searching the Web for medical information may be viewed as being on a quest for a second opinion. Doctors seem to be ambivalent to such patients. Some doctors oppose bringing outside information to them. As late as 2018, it was claimed that, by searching the Web, patients made decisions regarding their medical issues and resisted their doctor’s advice.^44^ On the other hand, the attitude of Dutch consultants toward patients who used the Web was moderately positive, even though about half of the patients had difficulties in remaining updated with reliable websites.^45^ Another UK survey revealed that primary care doctors experienced considerable apprehension when patients presented to them information from the Web because of fear of being perceived as ignorant; however, eventually, they learned to respond to such patients by delaying their response (*I am not familiar with this particular website. I would like to read it and put some thought to it*) or using the Web as an ally (*In the meantime*, *you may wish to look up the websites* …).^46^

Some patients refrain from communicating to their doctor the information being sought due to concerns that the doctor may be offended. Other patients seem to expect their doctor to discuss the Web information and offer his/her professional opinion.^36^ Evidence suggests that Web searches for medical information are not a threat to the doctor–patient relationship. One survey found that searching the Web for health information improved the doctor–patient interaction for 77% of patients. Most patients reported that they would never doubt their doctor’s diagnosis, or reduce adherence to treatment because of conflicting online information.^35^ However, a dismissive or patronizing doctor’s attitude was reported to harm the doctor–patient relationship, occasionally to the extent of patients changing doctors.^36^

## DISCUSSION

The main challenges to SDM implementation in clinical practice are overcoming time constraints and providing unbiased information.

With regard to time constraints, the described teaching approach may appear impractical to medical students in view of the restricted duration of visits to family doctors in Israel. However, SDM does not seem, in and of itself, to prolong unduly the length of the doctor–patient encounter. A 2019 literature review indicated that the median duration of consultations using patient decision aids was 24 (4–68) minutes, while that of usual care was 21 (4–66) minutes, a difference of 2.6 minutes.^2^ Fortunately, the vast majority of patients in the community do not require such a 21 minute “usual care” visit. Patients with chronic serious disorders, in whom SDM is anticipated to take longer, may have to overcome the limited duration of visits to family physicians, either by setting up special appointments, or by subdividing the SDM sequence into multiple shorter encounters. Even if future research indicates that SDM requires more time, it would still be worthwhile, as it avoids lawsuits, educates patients, and potentially saves time in future encounters.

The second professed barrier of implementing SDM relates to overcoming biases in communicating probabilistic information to patients. Evidence suggests that even educated persons may be confounded by data presented in different frames and sequences.^47^ However, this evidence has been derived from the responses of various study populations to hypothetical situations. These (fast/immediate) responses in experimental settings cannot be generalized over the (slow, unhurried, and thorough) deliberations during doctor–patient encounters in clinical settings. It stands to reason that the high-stakes decisions during SDM can be readily recognized as requiring slow thinking that would avoid the biases of fast/immediate responses in experimental settings.

However, the main challenge to teaching SDM is not how to overcome these two barriers, but, rather, how to make learners aware of the importance of understanding a specific patient’s concerns. First, gaining such insight is necessary in order for doctors to meet patients’ expectations and to address the specifics of each individual case.^24^ Second, a doctor’s insight into the patient’s concern*s* creates an atmosphere of trust (*The doctor understood how I was feeling*). Finally, it is also the beginning of the multiple-phase process of empathy,^22^ whereby awareness of patient concerns triggers emotional engagement, compassion, and the desire to help. The first step in this sequence, namely gaining insight into the patient’s concerns, is especially important, because failure to identify them precludes empathy. We believe that this first step is a teachable skill. It requires conducting a patient-centered interview, conveying sustained respect and interest, and using questions such as those suggested throughout this paper.

## Abbreviations

SDM: shared decision making
